# Expression of CCR6 in esophageal squamous cell carcinoma and its effects on epithelial-to-mesenchymal transition

**DOI:** 10.18632/oncotarget.23318

**Published:** 2017-12-15

**Authors:** Jian Liu, Xiao Zheng, Haifeng Deng, Bin Xu, Lujun Chen, Qi Wang, Qi Zhou, Dachuan Zhang, Changping Wu, Jingting Jiang

**Affiliations:** ^1^ Department of Tumor Biological Treatment, The Third Affiliated Hospital of Soochow University, Changzhou 213003, China; ^2^ Jiangsu Engineering Research Center for Tumor Immunotherapy, Changzhou 213003, China; ^3^ Institute of Cell Therapy, Soochow University, Changzhou 213003, China; ^4^ Department of Oncology, The Third Affiliated Hospital of Soochow University, Changzhou 213003, China; ^5^ Department of Pathology, The Third Affiliated Hospital of Soochow University, Changzhou 213003, China

**Keywords:** C-C motif chemokine receptor 6 (CCR6), esophageal squamous cell carcinoma (ESCC), lymph node metastasis, epithelial-to-mesenchymal transition (EMT)

## Abstract

Esophageal squamous cell carcinoma (ESCC) is the most common esophageal cancer associated with poor prognosis. We detected the expression of C-C motif chemokine receptor 6 (CCR6) and epithelial-to-mesenchymal transition (EMT) markers in esophageal tissues/cells, and evaluated the effects of CCR6 on ESCC cells proliferation, migration and invasion in response to C-C motif chemokine ligand 20 (CCL20) treatment. Our data showed CCR6 was highly expressed in ESCC cell lines (ECA-109 and TE-1), whereas kept in a low expression in normal cell lines HEEC (*P* < 0.001). CCL20 stimulus induced a significant decrease in the proliferation ability of ESCC (*P* < 0.05). The healing speed of CCL20 group was significantly higher than control in ECA-109 (*P* < 0.01), whereas significantly lower in αCCR6+CCL20 group than CCL20 group (*P* < 0.05).The number of cells permeabling through the polycarbonate membrane in CCL20 group was higher than control (*P* < 0.01). The cell number in αCCR6+CCL20 group was significantly reduced compared to CCL20 group in ECA-109 (*P* < 0.05). Moreover, after CCL20 stimulated in ECA-109, both mRNA and protein level of E-cadherin significantly decreased compared to control, while Vimentin was significantly higher. In αCCR6+CCL20 group, mRNA and protein level of E-cadherin significantly increased compared to CCL20 group, while Vimentin was much lower than CCL20 group. There was no significant difference in TE-1. In summary, high expression of CCR6 existed in the lymph node metastasis and TNM stage of ESCC. CCR6 play an important role in the regulation of tumor cell proliferation, invasion and migration. CCR6 may participate in regulating the occurrence of EMT in ESCC.

## INTRODUCTION

Esophageal cancer (EC) is one of the most common digestive tract malignant tumor in the world, attributing to 478 000 incidence (320 800 males and 157 200 females) and 375 000 deaths (253 800 males and 131 300 females) in China in 2015 [1]. The Type of EC in China mainly includes squamous cell carcinoma, adenocarcinoma or undifferentiated carcinoma. About 90% of EC in China is esophageal squamous cell carcinoma (ESCC), which has rapid progression, strongly local invasive, high rate of relapse and low survival [2]. Though the ESCC can be completely cured if diagnosed early, poor survival and increasing deaths of ESCC patients are mainly due to rapid progression and locoregional metastasis recurrence [3]. Therefore, finding and defining the relevant molecular marker signature on ESCC which dictates invasion and metastasis is important to improve the patient’s outcome.

Cancer metastasis occurs when genetically unstable cancer cells adapt to a tissue microenvironment that is distant from the primary tumor, is a multi-step process. The process of transition from epithelial cell change into mesenchymal phenotype cell, known as EMT, is closely related to the progress of tumor. EMT in tumor contains primary tumor cells lose the polarity of epithelial cells properties (strong adhesion, lamellar structure), and translate into mesenchymal cells capable of migration and invasion characteristics (no cell polarity, loss the close connection between cells) [4]. A variety of proteins involved in various adherens junctions and cytoskeleton reorganization are regulated in the process. It includes E-cadherin, Vimentin, N-cadherin, β-catenin and so on [5]. Solving factors that induce EMT would greatly help the development of diagnostic and prognostic markers, as well as better therapy for ESCC.

Many other studies have found that chemokine and chemokine receptor interaction participates in tumor metastasis process including ESCC [6–10]. Especially, the expressions of CCR6 and CCL20 are very high in many human tumors including ESCC [11, 12]. CCR6-CCL20 axis also has been shown important in many autoimmune [13] and inflammatory diseases [14–16]. In the present study, we find that high CCR6 expression is related to advance ESCC. Furthermore, we find the biological significance of CCR6-CCL20 axis in ESCC and also prove involvement of this axis in EMT, which is known to be involved in poor outcome of ESCC. But the molecular mechanisms for this relation remain elusive.

## RESULTS

### Expression of CCR6 in esophageal tissues and cell lines

**Figure 1:**
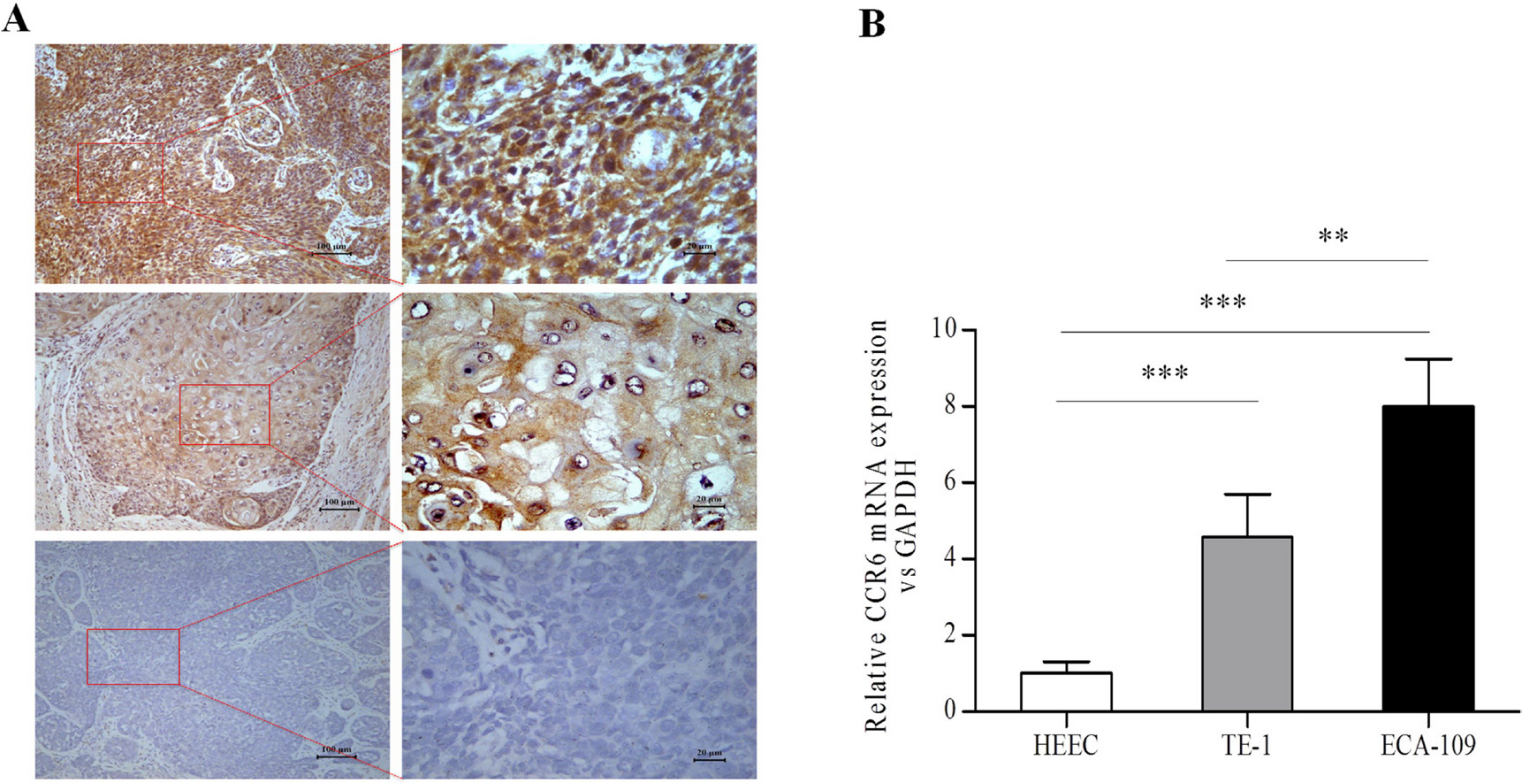
Analysis of CCR6 expression in esophageal tissues and CCR6 mRNA in esophageal cell lines (**A**) Immuno-intensity of CCR6 (brown) in ESCC tissues and normal esophageal tissue. Top two slides represent high immunological staining strength; in the middle two, the immune-staining intensity is moderate, and the bottom two are shown to indicate weak immune-staining. (**B**) CCR6 mRNA levels were significantly higher in ESCCcells (ECA-109, TE-1) compared to normal esophageal epithelial cells (HEEC). CCR6 mRNA was only expressed at a low level in HEEC. (^**^P < 0.01, ^***^P < 0.001).

Immunostaining of CCR6, E-cadherin and Vimentin were calculated as DAB-positive signals per mm^2^ of total membrane surface area and cytoplasm of tissue sections, the expression of CCR6 was significantly higher in cancer tissues compared with adjacent normal tissue (Figure 1A). Expression of CCR6 was higher in cases with lymph node-positive and high TNM stage compared with non-metastatic and low TNM stage cases (*P* < 0.05). Further, expression of E-cadherin was lower in cases with TNM high stage compared with TNM low stage (*P* = 0.001) (Table 1). Our data showed, the expression of CCR6, E-cadherin in esophageal squamous carcinoma with low correlation (*P* = 0.031), and no significant correlation between CCR6 and Vimentin expression (*P* = 0.492) (Table 2).

**Table 1:** Correlation of CCR6, E-cadherin and Vimentin expression with clinical data from ESCC patients

Parameters	CCR6	χ^2^	*P*	E-cadherin	χ^2^	*P*	Vimentin	χ^2^	*P*
	− +			− +			− +		
Gender Female Male	7 12 18 52	0.916	0.339	7 16 27 49	0.203	0.652	13 10 43 33	0.000	0.996
Age <60 ≥60	13 34 12 30	0.009	0.924	17 34 17 31	0.048	0.827	27 24 29 19	0.562	0.453
Tumor size (cm) <3.5 ≥3.5	13 22 12 42	2.340	0.126	11 24 23 41	0.204	0.652	18 17 38 26	0.582	0.446
T stage T1–T2 T3–T4	9 21 16 43	0.082	0.775	11 24 23 41	0.204	0.652	22 13 34 30	0.872	0.350
L-node metastasis Negative Positive	21 37 4 27	5.431	0.020^*^	25 40 9 25	1.423	0.233	39 26 17 17	0.909	0.340
Distance metastasis# Negative Positive	25 59 0 5	—	0.316	33 60 1 5	—	0.661	55 38 1 5	—	0.083
TNM stage I–I III–IV	21 35 4 29	6.621	0.010^*^	22 40 12 2	11.66	0.001^*^	35 27 21 16	0.001	0.976

CCR6, *n* = 89; E-cadherin and Vimentin, *n* = 99; Values in bold signify ^*^P < 0.05. #Fisher’s exact test.

**Table 2:** Correlation of the expression between CCR6, E-cadherin and Vimentin

			CCR6	E-cadherin	Vimentin
Spearman’s rho	CCR6	Correlation Coefficient	1.000	.224^*^	–.072
		Sig. (2-tailed)	.	.031	.492
		N	112	93	93
	E-cadherin	Correlation Coefficient	.224^*^	1.000	–.293^**^
		Sig. (2-tailed)	.031	.	.002
		N	93	105	105
	Vimentin	Correlation Coefficient	–.072	–.293^**^	1.000
		Sig. (2-tailed)	.492	.002	.
		N	93	105	105

^*^ Correlation is significant at the 0.05 level (2-tailed). ^**^Correlation is significant at the 0.01 level (2-tailed).

To further determine the expression of CCR6 mRNA in esophageal cell, two kinds of ESCC cell lines (ECA-109 and TE-1) and normal esophageal epithelial cell lines (HEEC) were subjected to qRT-PCR analysis of CCR6 mRNA. Similar to tissue expression, CCR6 mRNA levels were significantly higher in cancer cell lines compared to normal esophageal epithelial cell lines (*P* < 0.001). CCR6 was only expressed at a low level in HEEC (Figure 1B).

### CCR6-activation affects proliferation, migration and invasion in EC cells

**Figure 2:**
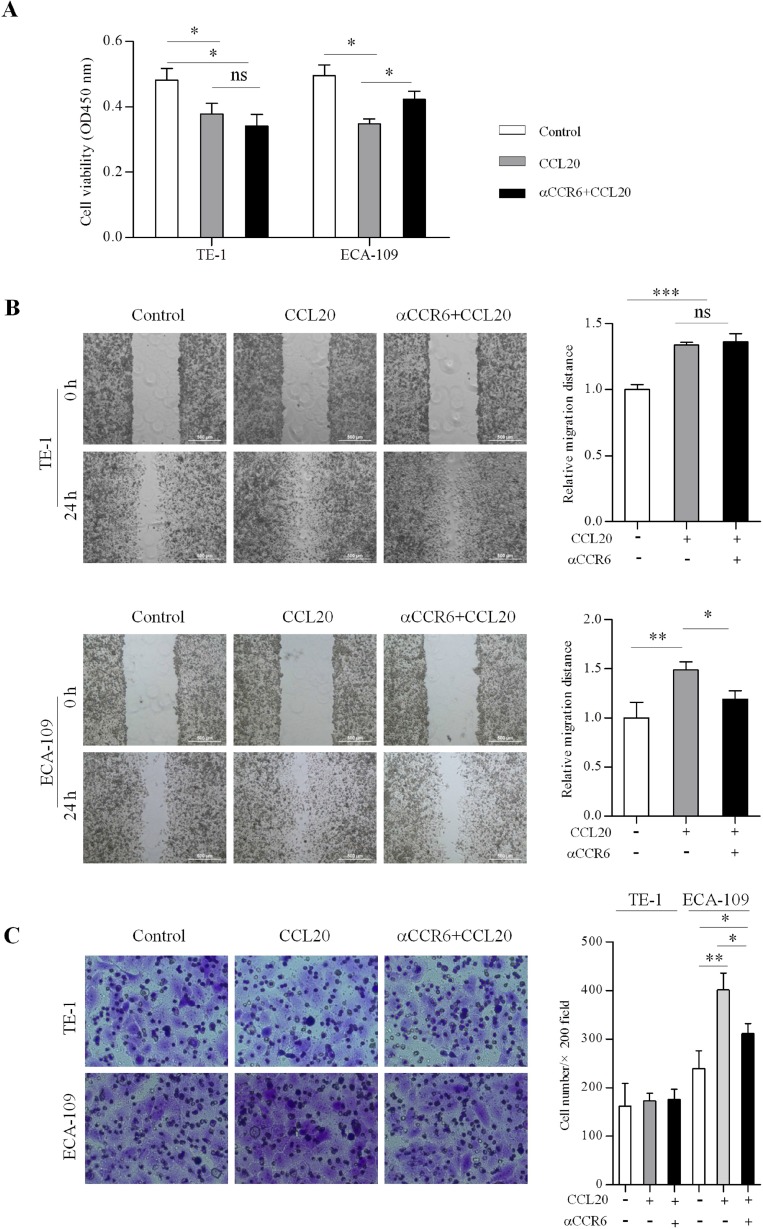
CCR6-activation affects proliferation, migration and invasion in ESCC cells (**A)** CCR6-CCL20 interaction inhibited proliferation of ESCC cells and promoted migration of ESCC cells. Proliferation of CCL20 treated and blocked CCR6 compared with untreated cells in ECA-109 and TE-1 cells after stimulated 24 hours are shown. (**B)** The healing speed of CCL20 treated and blocked CCR6 compared with untreated cells in ECA-109 and TE-1 cells after scratched 24 hours are shown. (**C)** ECA-109 cells showed higher invasive potential after CCL20 stimulated, compared to respective untreated cells and CCR6 blockade cells. Invasion was no significant difference in TE-1 cells. (^*^P < 0.05, ^**^P < 0.01, ^***^P < 0.001).

CCK-8 assay was used to determine proliferation in untreated and CCL20-treated EC cells. Proliferation of ESCC cell lines significantly decreased (*P* < 0.05) after CCL20 stimulated 24 hours compared with untreated samples. Proliferation ability increased significantly (*P* < 0.05) after blocking CCR6 in ECA-109 cells compared with CCL20 treated group (Figure 2A). The effect of CCR6-CCL20 axis on ESCC cell migration and invasion was characterised by wound healing and trans-well using CCL20 as a chemo-attractant. ESCC cell lines showed higher migratory potential toward CCL20 gradients, compared to respective untreated cells, which was significantly (*P* < 0.05) inhibited after CCR6 blockade in ECA-109 cells not in TE-1 cells (Figure 2B). In contrast, trans-well assay showed that treatment of TE-1 cells with CCL20 and blocking CCR6 did no noticeably alter cell invasion. There were significant difference in invasion between CCL20-treated and untreated cells (*P* < 0.01), also between CCL20-treated and anti-CCR6-treated (*P* < 0.05) in ECA-109 cells (Figure 2C).

### CCR6-CCL20 interaction affects EMT markers in EC cells

**Figure 3:**
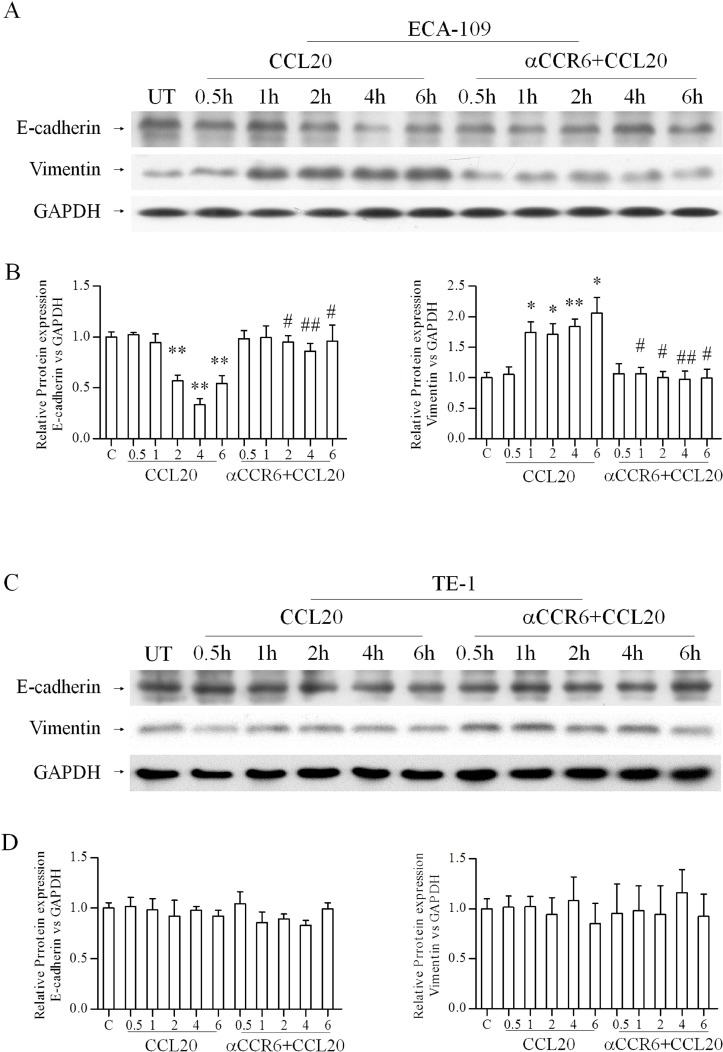
Effects of CCR6-CCL20 interactions on EMT of ESCC cell on protein level (**A**–**D)** The protein expression changes of E-cadherin and Vimentin in ESCC cell lines: ECA-109 (A, B), TE-1(C, D) after CCL20 treating and blocking CCR6 were showed. GAPDH was used as a loading control. (^*^P < 0.05, ^**^P < 0.01, ^#^*P* < 0.05, ^##^*P* < 0.01).

**Figure 4:**
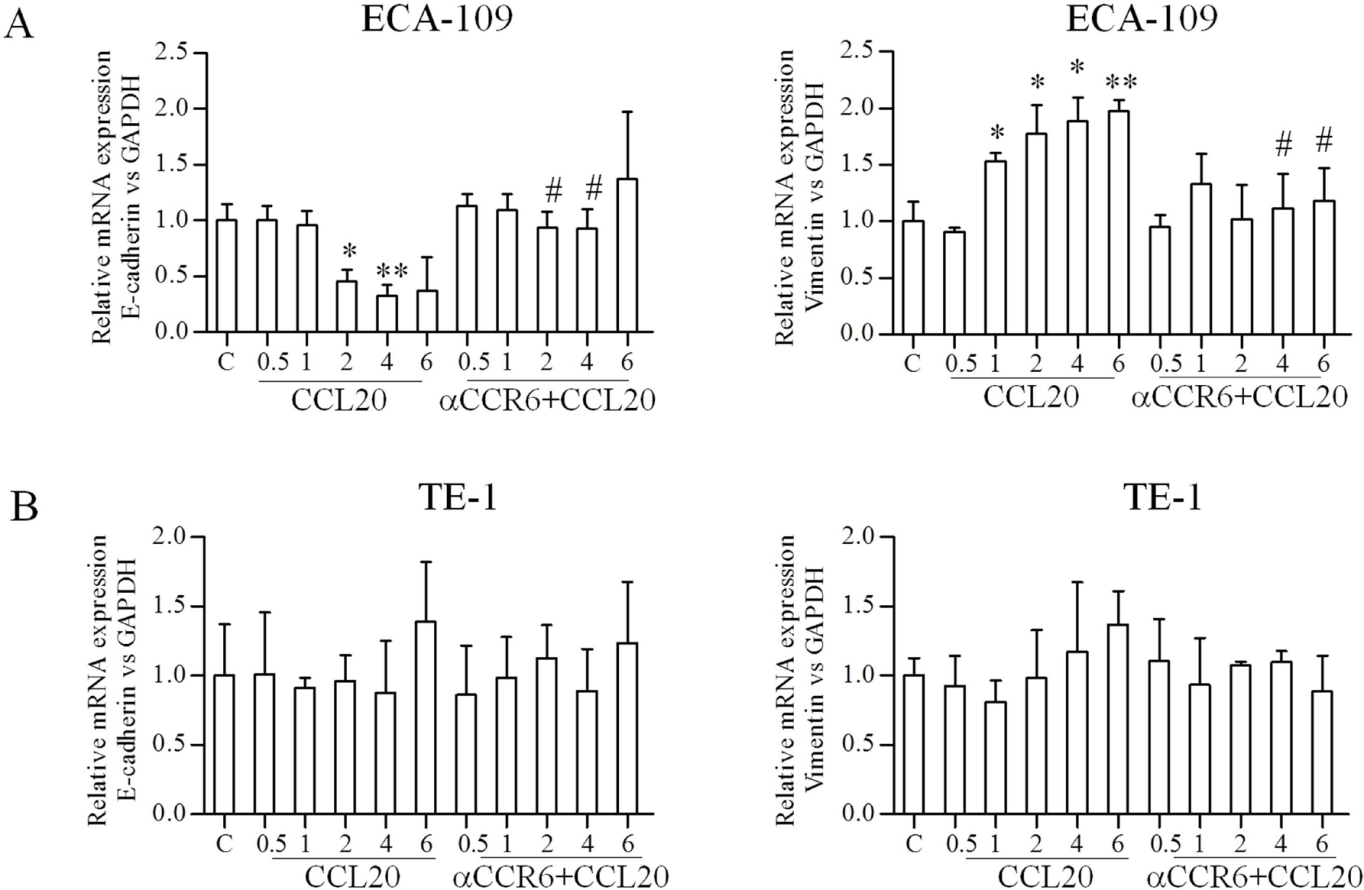
Effects of CCR6-CCL20 interactions on EMT of ESCC cell on mRNA level (**A**, **B**) QRT-PCR analysis was used to confirm E-cadherin and Vimentin protein expression results on mRNA level. GAPDH was used as a loading control. (^*^P < 0.05, ^**^P < 0.01, ^#^*P* < 0.05).

EMT promotes cancer cell metastasis and has a negative impact on disease progress and therapeutic outcome. Hence, we evaluated the effect of CCR6-CCL20 interaction on EMT markers (E-cadherin and Vimentin). Reduction in E-cadherin protein and increased in Vimentin protein were observed 1 hour after CCL20 treatment, meanwhile, an opposite results were observed after CCR6 blockade in ECA-109 cell lines, statistical significance of change in protein level of E-cadherin and Vimentin in CCL20 treated cells compared with untreated cells are indicated as ^*^P < 0.05, ^**^P < 0.01, and the change in protein level of E-cadherin and Vimentin in CCL20-treated cells compared with blocked CCR6 cells are indicated as ^#^*P* < 0.05, ^##^*P* < 0.01(Figure 3A–3B). Similar expression pattern after CCL20 treatment and blocked CCR6 were observed at mRNA level by qRT-PCR in ECA-109 cell lines (Figure 4A). EMT markers were not noticeably alter in TE-1 cell lines in either protein or mRNA level (Figures 3C–3DC, 4B).

## DISCUSSION

Cancer metastasis is a multiple and complex process. A variety of chemokine receptors such as CCR7, CCR2, CXCR4, have confirmed its important function in tumor invasion and metastasis [17–19] since Muller *et al*. [20] reported chemokine receptors were closely related to breast cancer metastasis. CCR6, an important CC subtype chemokine receptor, the main role of CCR6 protein in normal physiology is involved in host defense and the process of antigen presenting during immune response. It was recently confirmed to be associated with progression of various cancers [11, 21], including ESCC [22, 23]. Our immunohistochemistry results showed that the expression of CCR6 in ESCC tissues was higher compared with normal adjacent esophageal tissues. Overexpression of CCR6 in ESCC tissues strongly implies the potential function of CCR6 in metastasis mechanism of ESCC. Higher expression of CCR6 in lymph node-positive cases compared with N0 and higher expression in high TNM stage cases further suggests that CCR6 is a potential marker in ESCC aggressiveness. It can be used as a biomarker for diagnosis and treatment in ESCC. To further establish the biological function of CCR6 in ESCC, its mRNA expression was tested in ESCC cell lines and normal esophageal epithelial cell lines. Higher expression of CCR6 in cancer cells compared with normal cells confirms the clinical significance of CCR6 in ESCC at cellular level.

Abnormal proliferation of tumor cell promotes the progress of tumor, influence the curative effect of treatment. In this study, we found a significant inhibition in proliferation of cell lines (ECA-109 and TE-1) after CCL20 stimulated. This proliferation inhibition could be due to the cause of CCR6-CCL20 axis in promoting mesenchymal cell transition, which has weaker proliferation ability compared with epithelial cell [24, 25]. Cancer cells utilize kinds of migratory and invasive approaches in response to the cell external environment. Therefore, exploring the molecular determinants, which promote migratory and invasive of ESCC, can provide a new therapeutic option. Previous research showed that overexpression of CCR6 could promote the invasiveness of cancer cells by interacting with other tumorigenic factors [11, 23, 24]. Our vitro experiments demonstrated that higher migratory potential of EC cell lines in response to chemotactic gradient of CCL20 suggests a role of CCR6-CCL20 axis in ESCC cell migration, and emphasizes the potential of CCR6 as a therapeutic target. It also promoted the invasion of ECA-109 cell lines but not in TE-1 cell lines. Differences between the ESCC cell lines experimental results may be caused by cells differentiation degree and the different expression of CCR6.

Tumor cells need invasion, intravasation, migration and colonization to achieve metastatic goal. Interaction of various signaling pathways influences EMT that promotes these metastatic processes [26]. Chemokine receptor signaling make a significantly contribute in these processes [27, 28]. EMT is one of main methods to obtain the ability of invasion and metastasis in tumor cells. E-cadherin is a kind of epithelial calcium ions protein that across the membrane. It plays an important function in maintaining the organizational structures and cell polarity. Vimentin is a fibrous protein for maintaining the integrity of the cytoskeleton. Lower expression of E-cadherin and overexpression of Vimentin have a closely relationship with tumor progression [29, 30]. Hence, our western data showed decrease in E-cadherin and increase in Vimentin after CCL20 treatment in ECA-109 cell lines; meanwhile an opposite result of the E-cadherin and Vimentin expression were observed in ECA-109 cell lines after blocking the expression of CCR6. A consistent result was obtained by qRT-PCR detection in mRNA level. There were no significantly different changes in TE-1 cell lines both in protein and gene level. Our results were consistent with previous researches that CCR6 is associated with EMT in various cancers [23, 24, 31].

In conclusion, we found that overexpression of CCR6 in ESCC tissues and cell lines compared with normal adjacent tissue and normal esophageal epithelium cell lines. The differences of the CCR6 expression and the functional experimental study between esophageal cancer cell lines ECA-109 and TE-1 may be caused by the different degrees of differentiation in the ESCC cell lines. The expression of CCR6 in ESCC was significantly associated with nodal status and TNM stage. Inhibition of cell proliferation after CCL20 stimulated and activation of EMT markers, high migratory and invasive potential implicates association of CCR6-CCL20 with ESCC (ECA-109 cell lines) progresses. Although CCR6 and its ligand in the molecular mechanism of ESCC progress and function research is not clear. But with the deepening research of the CCR6 biology function, CCR6 and CCR6-CCL20 axis are expected to become new targets for treatment of ESCC, and provide a new strategy in inhibition of tumor growth and metastasis.

## MATERIALS AND METHODS

### Tissue specimens

Tissue microarray (TMA) was purchased from commercial source (Outdo Biotech Co. Ltd.). TMA contains EC tissue samples were obtained from 99 patients (76 males and 23 females, median age 59 years, range 37–78), who underwent esophageal resection for cancer with curative intent at the Department of Thoracic surgery, University of The Third Affiliated Hospital of Soochow between 2005 and 2006, and 11 cases of adjacent normal tissues. Clinical data was collected from the pathography of patients. All the clinical samples were approved by ethics committee of the third affiliated hospital of soochow university.

### Tissue chip

Read the H&E-stained histological slices of all ESCC cases again, and make marks in slices and the original wax blocks. Use 0.6 mm diameter suction needle suck organization from the wax block by tissue chip instrument, then loads in the position of receptor wax block according to the upfront design arrangement order. Drilled hole on the preparation blank paraffin block according to the number of sample and the required size of the organization, arranged in array. Successive 3μm slices lay on glass deal by Poly-L-Lysine after wax blocks were prepared.

### Immunohistochemistry

Esophageal TMA, containing cancer and normal adjacent tissues, were stained for CCR6, E-cadherin and Vimentin. Sections were deparaffinized three times for 15 min each in xylene. Sections were rehydrated for 5 min each in decreasing concentrations of ethanol (100%, 95% and 75%), followed by antigen retrieval in high temperature water, and washed in PBS three times. The sections were incubated with 3% H_2_O_2_ in PBS for 10 min for blocking endogenous peroxidase, rinsed three times with PBS, blocked with 3% BSA at RT for 20 min, and then incubated with primary antibodies at 4°C overnight in humidity chamber (E-cadherin, 1:500, MAB-0589, Maixin; Vimentin, 1:500, MAB-0178, Maixin; CCR6 antibody, 1:500, MAB195, R&D). Next, washed the sections with PBS and incubated with HRP-labeled goat anti-mouse/rabbit secondary antibody (KIT-5020, Maixin) for 1 hour at room temperature (RT). Diaminobenzidine was used as the chromogen and hematoxylin for the nuclear counterstain. Subsequently, the sections were dehydrated, cleared and mounted. The immunostaining intensity of CCR6 and E-cadherin were assessed according to the *H-score* method described by our previous report [32]: *H-score* = (% unstained × 0) + (% stained weak × 1) + (% stained moderate × 2) + (% stained strong × 3). The *H-scores* ranged from 0(100% negative) to 300(100% strong staining). For the evaluation of Vimentin staining, the rate of Vimentin positive cancer cells in all cancer cells was calculated and the positive rate was recorded.

### Cell culture and treatment

The esophageal cancer cell lines (ECA-109 and TE-1) and normal esophageal epithelial cell lines (HEEC) (The cell bank, Chinese academy of sciences) were used for study. Cell lines were cultured in RPMI 1640 (10% fetal calf serum, 100 U/ml penicillin, 100 μg/ml streptomycin) (Gibco). All cell lines were cultured at 5% CO_2_, 37°C.

### RNA extraction and real-time PCR

ESCC cell lines (2.5 × 10^6^/well) were seeded in 96 well plates. After overnight culture, cells were treated with CCL20 (100 ng/ml) for different time intervals (0.5, 1, 2, 4, 6) hours. Untreated cells were used as negative control. Total cellular RNA was extracted from cell lines using Trizol reagent according to the instructions of the manufacturer (Takara Bio). Nanodrop2000 was used to test the absorbance in 260 nm/280 nm, and then calculated concentration and purity, preserved in −80°C. cDNA was synthesized using GoScript-TM. Reverse Transcription system according to the manufacturer’s instructions (Promega). The relative expressions of EMT markers (E-cadherin and Vimentin), CCR6 and control GAPDH were continuously detected during 40 cycles by real-time PCR in 7500HT fast real-time PCR system (Applied Biosystems). Experiment was repeated three times. Specific primers and probes were obtained from Sangon Biotech. The sequences are in following Table 3.

**Table 3:** The sequences of PCR primers used in this study

Gene	Forward primer	Reverse primer
CCR6	5′-TTCAGCGATGTTTTCGACTCC-3′	5′-GCAATCGGTACAAATAGCCTGG-3′
E-cadherin	5′-CGAGAGCTACACGTTCACGG-3′	5′-GGGTGTCGAGGGAAAAATAGG-3′
Vimentin	5′-AGTCCACTGAGTACCGGAGAC-3′	5′-CATTTCACGCATCTGGCGTTC-3′
GAPDH	5′-TGTGGGCATCAATGGATTTGG-3′	5′-TGTGGGCATCAATGGATTTGG-3′

### Proliferation assay

ESCC cells were seeded at a density of 100 μl 1 × 10^4^/ well in 96 well plates, and then incubation at 5% CO_2_, 37°C overnight. Cells were stimulated with 100 μl recombinant CCL20 (360-MP-025, R&D) or αCCR6 (ab68131, Abcam) + CCL20, incubated 36 hours. Next, 10 μl CCK-8 reagent was added to the cells and incorporated CCK-8 was detected using Thermo Scientific Multiskan at A450 nm after 1 hour. Growth curve was drew with A450 average as the ordinate, time as the abscissa.

### Migration assay

Wound healing experiment was used for migration study. 96 well plates were packaged with FN (10 μg/μl) and incubation at 37°C overnight. Aspiration the solution and dry the plate in the next day. ESCC cells (5 × 10^5^) with or without blocking CCR6 used αCCR6 (1 μg/ml) and CCL20 (100 ng/ml) were added in the well with DMEM, and then incubated at 5% CO_2_, 37°C overnight. 1ml sterilization spear was scratched the bottom of culture plates vertically, uniformly and quickly. Took photos and measured the scratch width after 0 hour, 24 hours. According to the collected data analyzed the results and drew pictures.

### Invasion assay

Trans-well experiment was used for invasion study. ESCC cells (2 × 10^4^) with EMDM were added to the top chamber. DMEM with or without blocking CCR6 used 1 μg/ml αCCR6 and 100 ng/ml CCL20 were added in the bottom chamber. Cells were then allowed to invade under chemotactic of CCL20 at 5% CO_2_, 37°C overnight. Wiped out Non-invaded cells from top chamber with cotton swab. Cells at the bottom surface were fixed with 100% methanol for 20 min, stained for 2 hours with crystal violet, and rinsed three times with PBS. Invaded cells were counted under the microscope at 40 × magnification.

### Western blot assay

The relative levels of EMT markers (E-cadherin and Vimentin) were further confirmed by western blot analyses. ESCC cells were treated with 100 ng/ml CCL20, cell lysates were prepared using protease inhibitor cocktail containing RIPA buffer (Thermo Fisher Scientific, USA) at different time points (0.5, 1, 2, 4 and 6 hours) after CCL20 treatment. The control was the Proteins isolated from untreated cells. E-cadherin was resolved using 6% SDS-PAGE while Vimentin and GAPDH used 10% SDS-PAGE and transferred electrophoresis onto PVDF membrane. Used 5% skimmed milk to block the membranes for 2 hours at RT, and probed with primary anti-E-cadherin antibody (ab1416, Abcam), Anti-Vimentin antibody (ab92549, Abcam) at 4°C overnight. Membranes were incubated with peroxidase-conjugated anti-rabbit IgG secondary antibody (Beyotime) at RT for 1 hour. Immunoreactive protein bands were visualized with EZ-ECL Kit (Biolnd Biotech).

### Statistical analysis

All statistical analyses were performed by using SPSS 22.0 software (SPSS, Inc., Chicago, IL) and GraphPad Prism 5.0 software package (GraphPad Software, Inc., San Diego, USA). ‘*t*-test and oneway ANOVA were used to compare the means of two groups or more than two groups respectively, and Chi-square test was used to compare the ratio between different groups. Different groups of non-normal distribution data were compared by Kruskal-Wallis Test and Mann-Whitney *U* Test. The *P* value of less than 0.05 based on the two-sided test was considered to be statistically significant.

## Abbreviations

ESCC: Esophageal squamous cell carcinoma
EC: Esophageal cancer
CCR6: C-C motif chemokine receptor 6
CCL20: C-C motif chemokine ligand 20
EMT: Epithelial-to-mesenchymal transition
TNM: Tumor Node Metastasis
TMA: Tissue microarray
RT: Room temperature
FN: Fibronectin
DMEM: Dulbecco’s Modified Eagle’s Medium.
